# Prediction Models for COVID-19 Mortality Using Artificial Intelligence

**DOI:** 10.3390/jpm12091522

**Published:** 2022-09-16

**Authors:** Dong-Kyu Kim

**Affiliations:** 1 Institute of New Frontier Research, Division of Big Data and Artificial Intelligence, Hallym University College of Medicine, Chuncheon 24253, Korea; doctordk@naver.com; Tel.: +82-33-240-5180; 2 Department of Otorhinolaryngology-Head and Neck Surgery, Chuncheon Sacred Heart Hospital, Hallym University College of Medicine, Chuncheon 24253, Korea

**Keywords:** COVID-19, artificial intelligence, machine learning, prediction models, mortality, systematic review

## Abstract

The coronavirus disease 2019 (COVID-19) pandemic has placed a great burden on healthcare systems worldwide. COVID-19 clinical prediction models are needed to relieve the burden of the pandemic on healthcare systems. In the absence of COVID-19 clinical prediction models, physicians’ practices must depend on similar clinical cases or shared experiences of best practices. However, if accurate prediction models that combine parameters are introduced, they could provide the estimated risk of infection or experiencing a poor outcome following infection. The use of prediction models could assist medical staff in assigning patients when allocating limited healthcare resources and may enhance the prognosis of patients with COVID-19. Recently, several systematic reviews for COVID-19 have been published, some of which focus on prediction models that use artificial intelligence. We summarize the important messages of a systematic review titled “COVID Mortality Prediction with Machine Learning Methods: A Systematic Review and Critical Appraisal,” published in this Special Issue.

Coronavirus disease 2019 (COVID-19) is an infectious disease caused by the severe acute respiratory syndrome coronavirus 2 (SARS-CoV-2) that was discovered in China in December 2019 and declared a pandemic by the World Health Organization in March 2020. As of 7 September 2022, over 600 million confirmed cases of COVID-19, including almost 6.5 million deaths, were reported to the World Health Organization (WHO) since the start of the COVID-19 pandemic in late 2019 [1]. In the early phase of the COVID-19 pandemic, most healthcare providers had trouble with critical time-sensitive decisions, such as resource allocation, diagnosis, and treatment for patients with suspected SARS-CoV-2 infection because of the lack of robust evidence-based decision-support tools.

Artificial intelligence (AI) consists of the imitation of the cognitive functions and intelligent behavior of humans, performed by machines [2]. Machine learning is a subfield of AI that focuses on algorithms that enable computers to define a model for complex relationships or patterns from empirical data, without explicit programming. Deep learning is a subfield of machine learning that is more powerful and flexible than conventional machine learning algorithms and uses methods similar to biological neural networks to solve a wide variety of complex tasks. Thus, these approaches can be efficiently tested in healthcare applications such as disease diagnosis, analysis of medical images, big data collection, research and clinical trials, management of smart health records, and the prediction of outbreaks. Since the start of the COVID-19 pandemic, AI has been applied to the prediction of the COVID-19 trajectory and the development of diagnostic and prognostic models.

Previously, numerous studies for COVID-19 prediction models have been published using traditional statistics frameworks or AI algorithms. Additionally, several systematic reviews have shown the usefulness of prediction models for SARS-CoV-2 diagnosis and for prediction the disease severity, length of hospital stay, and the need for intensive care unit admission and mechanical ventilation [3,4,5,6]. However, systematic reviews on the prediction of COVID-19 mortality using AI technology are still lacking. An article by Bottino et al. [7], published in this Special Issue of the *Journal of Personalized Medicine*, reviews studies that used AI technology, including machine learning and deep learning, to predict COVID-19 mortality. The article provides possible explanations of the best results that the studies obtained. The review also discusses the limitations of current studies and provides suggestions for future research. To our knowledge, this article is the first systematic review of COVID-19 mortality prediction using AI technology.

This review includes 24 studies, of which 3, 17, and 4 studies used deep learning, traditional machine learning, and hybrid methodologies, respectively. All the studies included in the review considered the clinical characteristics, but only one study considered the computed tomography imaging characteristics. Most studies had an imbalanced number of patients who survived and who died. Additionally, both binary and multi-level characteristics were considered. The review summarizes the models developed according to the data source, data partitioning, class of features, machine-learning methodologies, and evaluation metrics for performance assessment, and focuses on mortality prediction using machine-learning techniques to fit nonlinear and complex interaction effects between predictors.

Collectively, this review identified some best practices that studies could follow for developing optimal machine-learning models. First, the use of a high-quality dataset with a large sample size, and a balanced number of individuals in each group are important for achieving good model performance. For these reasons, using a multi-country database is useful for obtaining generalizable results and determining the most important features because mortality cases in patients with COVID-19 are a relative minority among overall infected cases. Thus, it could induce data imbalance. Second, the existence of missing values is a challenging problem for the development of the prediction model. It means that some variables deleted during data curation could reduce the model performance. Thus, to overcome this issue, prospective multi-center studies are needed. Third, use of a combination of different machine-learning methodologies is highly effective. Of previously developed prediction models, those that use a combination of methods, such as ensemble models, tend to show the best performance. Fourth, clinical features should include several different types of characteristics, including demographics and laboratory test results. Multiple studies have shown a highly significant association of age and C-reactive protein and lactate dehydrogenase levels with COVID-19 mortality. Fifth, as many metrics as possible should be reported to have a complete view of model performance. Thus, model performance should be described using not only the most common metrics, such as area under the receiver-operating characteristic curve and accuracy, and other metrics for performance prediction assessment, including sensitivity, specificity, positive predictive value, and negative predictive value. Finally, real-time updates of prediction models for COVID-19 mortality are necessary because the COVID-19 pandemic is still evolving.

In conclusion, predicting the mortality of patients with COVID-19 is crucial for optimized clinical care and resource management during the pandemic period. AI-based technologies have played an important role in the prediction of COVID-19 mortality, and several healthcare centers have adopted and customized these technologies in response to the challenges posed by the COVID-19 pandemic. This systematic review may stimulate the development of improved AI models to predict mortality in patients with COVID-19.

## Data Availability

Not applicable.
